# Reducing fear and avoidance of memory loss improves mood and social engagement in community-based older adults: a randomized trial

**DOI:** 10.1186/s12877-023-04470-4

**Published:** 2023-11-29

**Authors:** Francesca R Farina, John Regan, Melissa Marquez, Hosanna An, Patricia O’Loughlin, Pavithra Pavithra, Michelle Taddeo, Rachel C Knight, Marc Bennett, Bert Lenaert, James W Griffith

**Affiliations:** 1 Department of Medical Social Sciences, Feinberg School of Medicine, Northwestern University, Chicago, IL 60611 USA; 2 Global Brain Health Institute, Trinity College Dublin, Dublin, Ireland; 3 School of Psychology, Trinity College Dublin, Dublin, Ireland; 4 School of Psychology, University College Dublin, Dublin, Ireland; 5 School of Nursing, Trinity College Dublin, Dublin, Ireland; 6 MRC Cognition and Brain Sciences Unit, University of Cambridge, Cambridge, UK; 7 Faculty of Psychology, Open University, Heerlen, The Netherlands; 8 Department of Neuropsychology and Psychopharmacology, Faculty of Psychology and Neuroscience, Maastricht University, Maastricht, The Netherlands

**Keywords:** Fear, Avoidance, Memory loss, Alzheimer’s Disease and its related Dementias, Older adults, well-being.

## Abstract

**Background:**

Alzheimer’s disease and related dementias (ADRD) are among the most feared age-related conditions. The aim of this study was to evaluate a brief psychological intervention to promote adaptive coping in older adults experiencing heightened fear of ADRD and investigate positive downstream effects on health-related secondary outcomes, including frequency of reported memory failures, psychosocial functioning, and quality of life.

**Methods:**

Eighty-one older adults were recruited and randomized into REFRAME or active control intervention arms. Both groups received psycho-education and training in mindful monitoring of fears related to ADRD. The REFRAME group received an additional behavioral activation component intended to disrupt maladaptive avoidant coping (i.e., avoidance) strategies. Both groups completed 3-weeks of intervention exercises with accompanying questionnaires (baseline, mid- and post-intervention and 4-week follow-up).

**Results:**

Adherence was strong (> 75%). We observed a significant reduction in ADRD-related fear and avoidance in both groups. Significant reductions were also observed for frequency of self-reported memory failures, anxiety, and depression. Depression was significantly reduced in the REFRAME group compared to the control group. Significant increases in participants’ ability to participate in social activities and well-being were also observed.

**Conclusions:**

Findings suggest that a brief psychological intervention can mitigate ADRD-related fears and avoidant coping in older adults, and that benefits extend to broader health-related outcomes including anxiety, depression, social functioning, and well-being. Addressing ADRD-related fear has implications for healthy aging and risk reduction, as individuals may be more likely to engage in activities that are protective against ADRD but were previously avoided.

**Trial registration:**

: https://clinicaltrials.gov/ct2/show/NCT04821960.

**Supplementary Information:**

The online version contains supplementary material available at 10.1186/s12877-023-04470-4.

## Background

More than 6 million older Americans have Alzheimer’s disease (AD), the most common form of dementia [1]. This number is expected to double by 2060 [1]. As the prevalence of AD and related dementias (ADRD) increases, so too does awareness and anxiety around these conditions [2–4]. Fear of ADRD is common in the general population and highest among older adults [5–7]. Survey evidence indicates that people aged 50+ years are more fearful of ADRD than cancer, stroke, and heart conditions [8]. Fears typically focus on memory loss as an early and well-recognized symptom of ADRD [9].

Many factors can drive fear of ADRD, including age, perceived own risk for developing ADRD (e.g., due to genetics or family history), and being less knowledgeable about ADRD [10–12]. Common fears include loss of self-identity, independence, and dignity, becoming a burden to others, and long-term care [10, 13]. Depictions of ADRD in literature and media also shape societal perceptions, which can, in turn, influence fear and stigma. ADRD depictions in popular culture are predominantly negative, with dementia being framed as an epidemic or war, people with dementia being ‘the living dead’ and the burden of care [14].

Experiencing high levels of fear around ADRD is associated with poorer health outcomes. Specifically, heightened ADRD-related fear has been linked to higher psychological distress, increased frequency of self-reported memory failures, and poorer quality of life in middle-aged and older adults [9, 15, 16]. Over time, heightened fear can also result in avoidant coping strategies that mitigate short-term distress but come at a long-term cost [2, 14, 17]. In the context of ADRD, individuals who are highly fearful may delay screening or support-seeking, thus undermining opportunities for early screening and intervention [18].

Individuals who are highly fearful of ADRD may also withdraw from socially or cognitively demanding activities, thus undermining opportunities for to maintain social connection and build cognitive reserve. This pattern of fear-avoidance behaviors has been demonstrated in the fear of falling literature, whereby individuals who experience a fall begin to avoid movements or activities based on the fear of (re-)injury [19]. Crucially, this literature shows that fear can develop in the absence of any direct negative outcome, i.e., a fall, or in the case of ADRD, a memory lapse. Fears can emerge indirectly through social observation or verbal instruction [19–21].

How individuals cope with fears about ADRD therefore has potential to influence behavioral choices, which could impact lifestyle-related risk, for example through low mood and social isolation. Despite this, however, no studies have evaluated psychological interventions aimed at disrupting patterns of avoidant coping brought on by heightened fear specific to ADRD symptoms. Previous interventions have mainly focused on improving the psychological well-being of carers and people with ADRD [22]. Interventions in community-based older adults have focused on reducing worry (through group cognitive behavioral therapy) and fear of ADRD (through an Alzheimer’s disease knowledge training program) [23, 24].

The Reducing Fear and Avoidance of Memory Loss (REFRAME) study was designed as a randomized control trial (RCT) with the goal of promoting adaptive coping in community-based older adults experiencing heightened fear and avoidance around ADRD [25]. The trial consisted of two intervention arms: (1) REFRAME group who received psycho-education, training in mindful awareness of fears, and behavioral activation (i.e., promoting engagement in behavioral and social activities), and (2) active control group who received psycho-education and mindful awareness only. The inclusion of a behavioral activation component in the REFRAME arm was hypothesized to provide additional benefits above and beyond psycho-education and mindfulness by intentionally disrupting patterns of avoidant behavioral coping [25]. The intervention was specifically targeted at older adults experiencing heightened fear in the absence of cognitive impairment – sometimes referred to as the “worried well”. The term “worried well” is used to describe individuals who are concerned that they may have (or be developing) dementia, who are neurological normal and whose neuropsychological profile is within expected limits for their demographic profile [26].

The primary aim of the current study was to evaluate if the REFRAME intervention could promote adaptive coping strategies in older adults; namely, mindful awareness of fears about ADRD and a tendency towards engaging in cognitively and socially stimulating activities [25]. Additionally, we sought to evaluate if the intervention had positive downstream effects on health-related secondary outcomes, including frequency of reported memory failures, psychosocial functioning, and quality of life. We hypothesized that both the REFRAME and active control intervention arms would be effective at reducing ADRD-related fear, but that the positive effects would be stronger in the REFRAME group. We further hypothesized that both groups would show positive effects on secondary outcomes, including reductions in frequency of memory failures, and increases in psychosocial functioning and well-being. Again, we anticipated the positive effects to be stronger in the REFRAME group.

## Methods

### Study design and participants

The REFRAME study was an RCT conducted at Northwestern University, Chicago, IL, USA. Participants were recruited from the community using advertising (e.g., flyers, email outreach, postings on transit lines) and through official registries (e.g., ResearchMatch). Inclusion criteria were: ≥ 55 years of age, literate in English, resident in Illinois, elevated fear and avoidance of memory loss defined as a score of > 60 (75% percentile) on the Fear and Avoidance of Memory loss scale [11, 16], able and willing to provide informed consent, willing to be randomized to intervention group and study measures, and access to the internet to engage with materials. Exclusion criteria were: diagnosis of Mild Cognitive Impairment or ADRD, cognitive impairment defined as a score of < 18 on the Montreal Cognitive Assessment Blind version (MoCA-Blind) [27], severe depression defined as a score of ≥ 12 on the 15-item Geriatric Depression Scale (GDS-15)” [28], history of hospitalization in the 6 weeks previous to the study or repeated emergency room visits, current psychotherapy treatment, current substance use disorder, and inadequate vision or hearing to engage with study materials.

The study was carried out in accordance with the Code of Ethics of the World Medical Association (Declaration of Helsinki). Informed consent was obtained from all participants prior to beginning the study. The study was approved by the Institutional Review Board at Northwestern University, Chicago, in March 2021 (ref: STU00214078). The study is reported according to CONSORT reporting guidelines.

### Randomization

Participants were randomized into the REFRAME or active comparison group using allocation sequence created in the blockrand package of R [29], stratified by sex, by members of the research team.

### Procedures

**Fig. 1:**
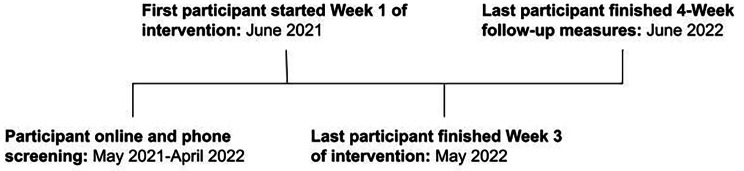
Study timeline

Participants completed screening measures at baseline, and outcome measures at baseline, Weeks 1–3 of the intervention, 1-week post-intervention, and 4-week follow-up (Table 1). Due to the COVID-19 pandemic, the intervention protocol was adapted to facilitate remote completion via telephone and online using the REDCap platform [30]. The study timeline is shown in Fig. 1.

***Screening.*** Demographic profile was obtained for all participants at screening, including date of birth, sex, race, ethnicity, educational attainment, employment, income, and family history of ADRD. Participants also completed the GDS-15, FAM scale and the Fear of Alzheimer’s disease Scale (FADS) [31]. Participants who were eligible for the study at this point completed the MoCA-Blind over the phone, administered by a trained member of the research team. Those who were still eligible were randomized into an intervention arm.

**Table 1:** Summary of measures administered

Measures	Baseline	Intervention (weeks)			Post-intervention	Follow-up
		1	2	3		
Montreal Cognitive Assessment for blind individuals (MoCA-Blind)	X					
Geriatric Depression Scale (GDS-15)	X					
Fear and Avoidance of Memory Loss (FAM) scale	X	X	X	X	X	X
Fear of Alzheimer’s Disease Scale (FADS)		X			X	X
Memory Failure Scale (MFS)		X			X	X
Patient-Reported Outcomes Measurement Information System Profile (PROMIS-29)		X			X	X
World Health Organization-Five Well-Being Index (WHO-5)		X				X
Coronavirus Anxiety Scale (CAS)	X	X				X

***Intervention.*** Full details of the intervention protocol have been published previously [25], and the trial is registered on ClinicalTrials.gov (ID: NCT04821960, date: 30/03/2021). Participants provided self-reported information on psychological and health-related factors (see § 2.4; Table 1). Each week, participants received a link to the intervention materials for that week to complete on their own at home. Participants were instructed to complete the materials in a quiet environment with minimal distractions.

The intervention comprised three components, divided into 4–5 modules per week, and delivered as written text or audio, with accompanying online exercises (Appendix 1). Week 1 focused on psycho-education around ADRD and its symptoms, everyday memory lapses, and fear of ADRD in the population (e.g., prevalence). Week 2 focused on mindfulness training, including an introduction to the concept of mindfulness, meditations (e.g., the body scan), mindful monitoring of fearful thoughts and sensations, and psychological grounding. All participants completed the same Week 1 and 2 exercises. Week 3 for the REFRAME group focused on behavioral activation, including an introduction to avoidance and safety behaviors, and techniques for overcoming avoidance. Week 3 for the active control group focused on continued mindfulness training, including novel exercises that were thematically similar to Week 2. The intervention was self-directed; that is, participants were free to complete the modules in one sitting or spread out, depending on their preference. Each module took 20–30 min to complete, totaling 1–2 h of content per week.

### Outcome measures

***Primary outcome.*** ADRD-related fear and avoidance was assessed using the FAM scale, which focuses on the symptom of memory loss [11, 16]. The FAM is an 18-item self-report scale assessing fears and avoidance behaviors associated with memory loss, which has been validated for use in older populations [11, 16]. Items address two components: fear (e.g., “*I am afraid that I might embarrass myself by forgetting something*”) and avoidance (e.g., “*I try not to exert my brain too much as it might make my memory worse*”). Higher scores indicate greater fear and avoidance of memory loss (range = 18–90).

***Secondary outcomes.*** Fear of ADRD more generally was assessed using the Fear of Alzheimer’s Disease Scale (FADS), which focuses on fear of AD [31]. The FADS is a 30-item self-report scale validated for use in older adults. Higher scores indicate greater fear of developing AD. Everyday memory failures were assessed using the Memory Failures Scale (MFS) [32]. The MFS is a validated 12-item self-report scale. Higher scores indicate greater frequency of memory failures experienced in everyday life. Anxiety, depression, and ability to perform social roles and activities, were assessed using the Patient-Reported Outcomes Measurement Information System 29-item profile (PROMIS-29) scales [33]. The PROMIS-29 is a validated self-report scale assessing multiple health domains, including anxiety, depression, and ability to perform social roles and activities. Each domain is assessed using a 4-item subscale [33]. Higher scores indicate higher anxiety, depression, and ability to perform social roles, respectively. The PROMIS-29 also includes subscales for fatigue, sleep disturbance, physical function, pain intensity, and pain interference; these were not examined here. Well-being was assessed using the World Health Organization-Five Well-Being Index (WHO-5) [34]. The WHO-5 is a validated 5-item self-report scale assessing well-being over the last two weeks. Higher scores indicate greater well-being.

***Participant feedback***. Following the intervention, participants answered debriefing questions. Questions focused on aspects they found most helpful, challenges related to completing the intervention, and recommendations for improvement.

### Statistical analysis

We aimed to recruit 80 participants (n = 40 per group). Group sizes are powered to observe a significant interaction between time (within-person) and treatment (between-group) using a mixed model. Assuming a medium-sized (d = 0.4), group sizes of 21 are powered at 80% to observe a significant group-by-time interaction (alpha = 0.05, calculated using G*Power v3.1.9.7). Analyses were completed in RStudio v2022.02.3 [35]. The alpha level was set at 0.05. Primary and secondary outcomes were analyzed using linear mixed effects models using the lme4 package [36, 37]. Changes in each outcome measure were analyzed using full and reduced models. Full models included a random intercept for each participant, fixed main effect for group (1 = REFRAME, 0 = control), dummy variables for each time point, and group-time interactions for time points after the groups diverged. This allowed us to model the impact of treatment for each time point where the groups completed different intervention materials.

For each outcome, the full model was compared to a reduced model, which omitted any effects of group or group-time interactions. Reduced models included a random intercept for each participant and dummy variables for time only. This allowed us to model the impact of treatment for each time point regardless of group. Full and reduced models were compared using Analysis of Variance (ANOVA). In cases where the models did not differ (i.e., in variability explained), results from the reduced models are presented only.

Age was included as a covariate in all models. Given the broader study context, we repeated our analyses with the Coronavirus Anxiety Scale [38] as an additional covariate. Participant feedback was analyzed by qualitatively grouping the data and generating common themes.

## Results

### Participants

**Fig. 2:**
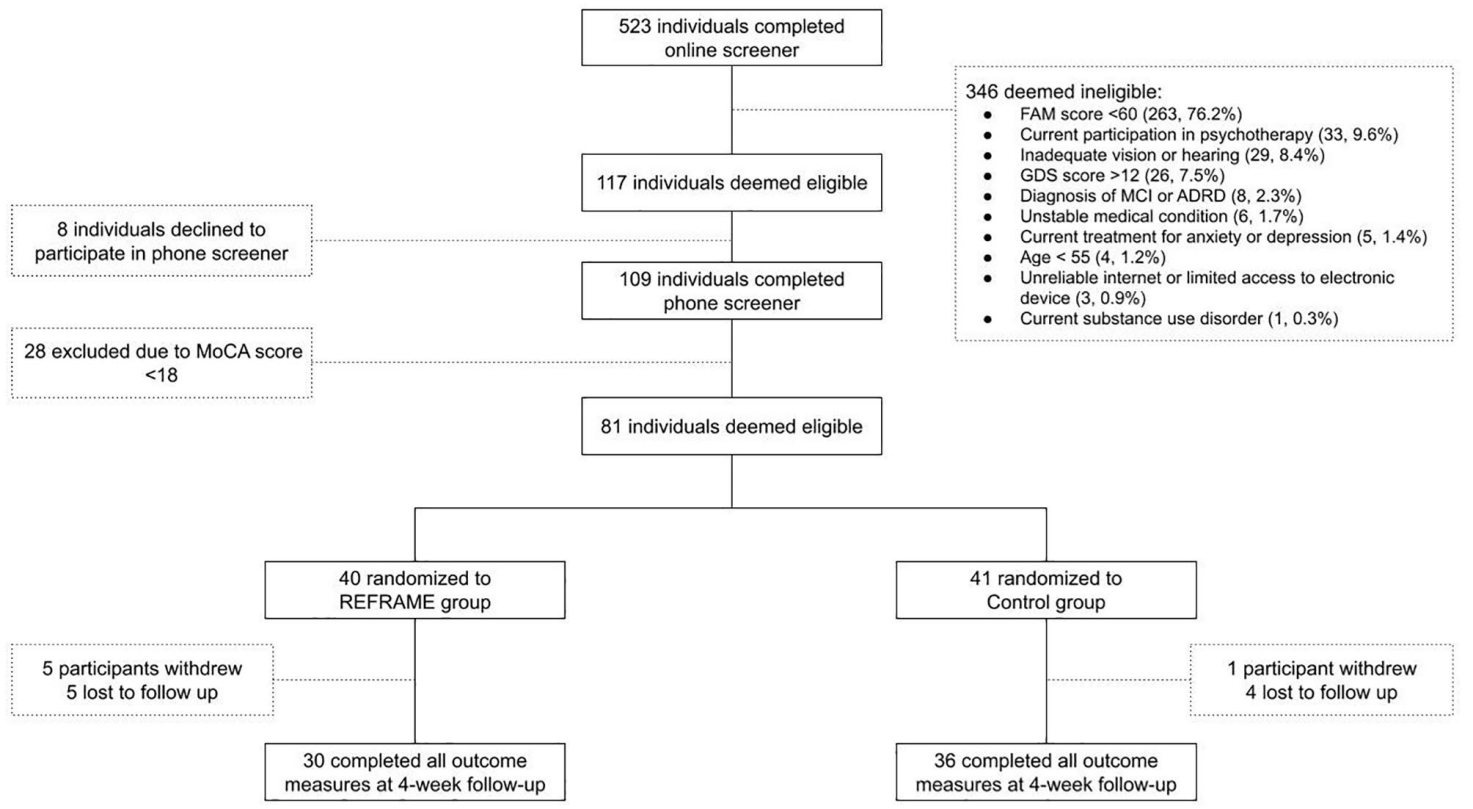
Recruitment flowchart. Abbreviations: ADRD = Alzheimer’s disease and its related dementias; FAM = Fear and Avoidance of Memory loss scale; GDS = Geriatric Depression Scale; MCI = Mild Cognitive Impairment; MoCA-Blind = Montreal Cognitive Assessment (version for the blind, which can be administered by phone)

Eighty-one participants were recruited between May 2021 and June 2022 and randomized, 40 into the REFRAME group and 41 into the active control group. All analyses were completed according to original assigned group. Mean age of the sample was 65.4 (± 7.1 years) and 71.6% (N = 58) identified as women (Table 2). Sixty-six participants completed the study in full, 30 in the REFRAME group and 36 in the control group (Fig. 2). The difference in completion rates across groups was not significant (χ^2^ = 2.31, *p* = .197, φ = 0.029). Of those who did not complete the study in full, the most frequently reported reason for withdrawal was not having sufficient time to engage with the materials. Demographic differences between participants who completed the study and those who did not were not significant (Table S1, Appendix 1). Engagement was high, with participants completing 75.5% of module materials. Engagement with study materials decreased over time (i.e., 83% on week 1 vs. 68.1% on week 3, B = -13.7, *p* = .013).

**Table 2:** Participant Demographics by Intervention Group

	REFRAME (n = 40)	Control (n = 41)	Total sample (N = 81)
Demographics			
Age (years ± SD)	67.0 (7.1)	63.7 (6.8)	65.4 (7.1)
Sex			
Number of females (%)	29 (72.5)	29 (70.7)	58 (71.6)
Number of males (%)	11 (27.5)	12 (29.3)	23 (28.4)
Race: number identifying as White (%)	32 (80)	39 (95.1)	71 (87.7)
Ethnicity: number identifying as non-Hispanic (%)	39 (97.5)	41 (100)	80 (98.8)
Education: number with a college degree or higher (%)	26 (65.0)	35 (85.3)	61 (75.3)
Employment: number retired (%)	19 (47.5)	21 (51.2)	40 (49.4)
Income: number reporting high income (%)	15 (37.5)	15 (38.5)	30 (38.0)
Number reporting family history of ADRD (%)	19 (47.5)	19 (46.3)	38 (46.9)

Notes: High income was defined as an average yearly household income of $75,000 or above. Participants who identified as non-White included the following categories: Black (N = 8; 9.9%), American Indian or Alaska native (N = 1; 1.2%), and Asian (N = 1; 1.2%)

### Efficacy

Table 3 presents scores for each outcome measure by intervention group and overall.

**Table 3:** Participant Scores on Questionnaires by Intervention Group

	REFRAME (n = 40)	Control (n = 41)	Total sample (N = 81)
Cognition (mean MoCA-Blind ± SD, possible range = 0–22)	19.7 (1.2)	19.9 (1.3)	19.8 (1.3)
Depression (mean ± SD, possible range = 0–15)	3.2 (2.4)	3.7 (2.7)	3.5 (2.6)
Fear-avoidance of memory loss (mean ± SD, possible range = 18–90)	67.8 (5.4)	68.0 (5.2)	67.9 (5.3)
Fear of Alzheimer’s disease (mean ± SD, possible range = 30–120)	45.2 (20.0)	47.3 (20.7)	46.3 (20.3)
Memory failures (mean ± SD, possible range = 12–60)	32.2 (8.5)	34.9 (7.7)	33.7 (8.2)
Patient-reported outcome subscales			
Anxiety (mean T score ± SD, possible range = 40.3–81.6)	55.2 (8.4)	55.1 (7.0)	55.1 (7.6)
Depression (mean T score ± SD, possible range = 41.0-79.4)	49.8 (8.1)	50.8 (9.1)	50.3 (8.7)
Social function (mean T score ± SD, possible range = 29.0-64.1)	52.1 (8.2)	52.1 (8.3)	52.1 (8.2)
Well-being (mean ± SD, possible range = 0–25)	14.2 (5.8)	13.9 (5.7)	14 (5.7)
Coronavirus Anxiety Scale (mean ± SD, possible range = 0–3)	1.8 (3.3)	0.7 (1.4)	1.2 (2.5)

Questionnaires include the range of possible scores. Abbreviations: MoCA-Blind = Montreal Cognitive Assessment for those who are visually impaired. Notes: values represent baseline scores for fear and avoidance of memory loss, MoCA-Blind, depression and Coronavirus anxiety, Week 1 scores for fear of Alzheimer’s disease, memory failures, general anxiety, depression, ability to participate in social activities (i.e., social function), and well-being

**Fig. 3:**
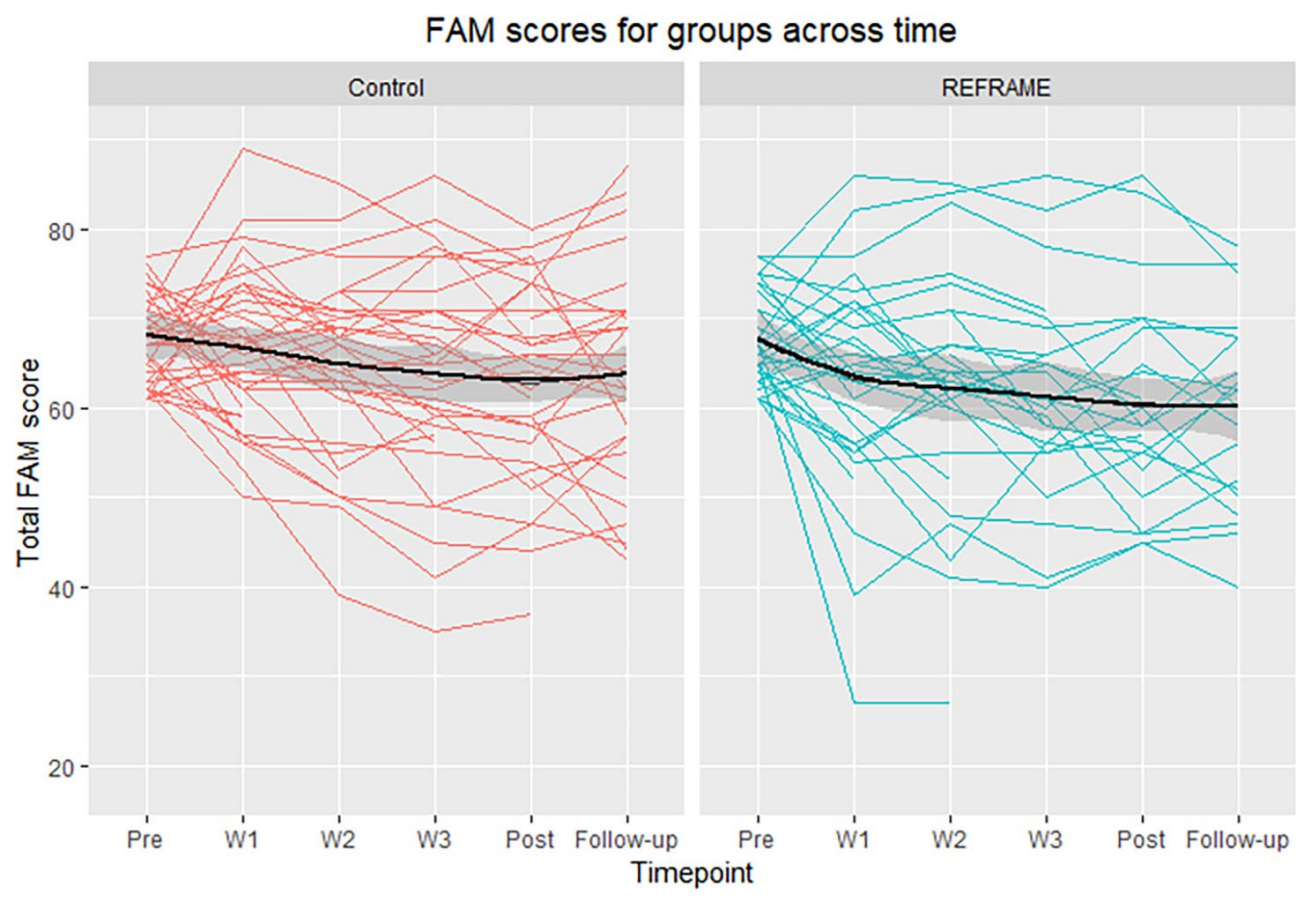
Graph of individual FAM scores for each participant across the six timepoints (pre-intervention, Weeks 1–3, post-intervention, and follow-up) in Control (red) and REFRAME (blue) groups. Colored lines indicate individual participant scores. Black lines represent group means, with surrounding gray representing the LOESS smooth curve with 95% confidence interval

***Primary outcome.*** FAM scores decreased regardless of intervention group. That is, including group and group-time interactions (full model) did not explain more variability compared to the time only (reduced) model (χ^2^ = 4.31, *p* = .365). Relative to baseline, FAM scores were significantly reduced across all time points, including 4-week follow-up (B = -6.63, *p* < .001, 95% confidence intervals (CI) = -8.60, -4.65; Fig. 3). On average, scores decreased by 5.6 points from baseline to follow-up. These effects remained when Coronavirus anxiety was added to the model (Table S2, Appendix 2).

***Secondary outcomes.*** FADS scores decreased regardless of intervention group. That is, including group and group-time interactions did not improve model performance (χ^2^ = 1.58, *p* = .665). Relative to baseline, FADS scores were significantly reduced across all time points, including follow-up (B = -5.34, *p* = .001, 95% CI = -8.53, -2.13). On average, scores decreased by 3.8 points from baseline to follow-up. These effects remained when Coronavirus anxiety was added to the model (Table S2).

MFS scores decreased regardless of group. Including group and group-time interactions did not improve model performance (χ^2^ = 5.31, *p* = .150). Relative to baseline, MFS scores were significantly reduced across all time points, including follow-up (B = -1.63, *p* = .008, 95% CI = -2.80, -0.46). On average, scores decreased by 1.3 points from baseline to follow-up. These effects remained when Coronavirus anxiety was added (Table S2).

Anxiety scores decreased regardless of group. Including group and group-time interactions did not improve model performance (χ^2^ = 2.61, *p* = .457). Relative to baseline, anxiety scores were significantly reduced across at follow-up (B = -1.64, *p* = .039, 95% CI = -3.17, -0.10). On average, scores decreased by 1.2 points from baseline to follow-up. Ability to participate in social activities scores increased regardless of intervention group. Including group and group-time interactions did not improve model performance (χ^2^ = 1.47, *p* = .690). Relative to baseline, ability to participate in social activities scores were significantly higher across all time points, including follow-up (B = 2.34, *p* = .007, 95% CI = 0.66, 4.01). On average, scores increased by 2.2 points from baseline to follow-up. These effects remained when Coronavirus anxiety was added (Table S2).

**Fig. 4:**
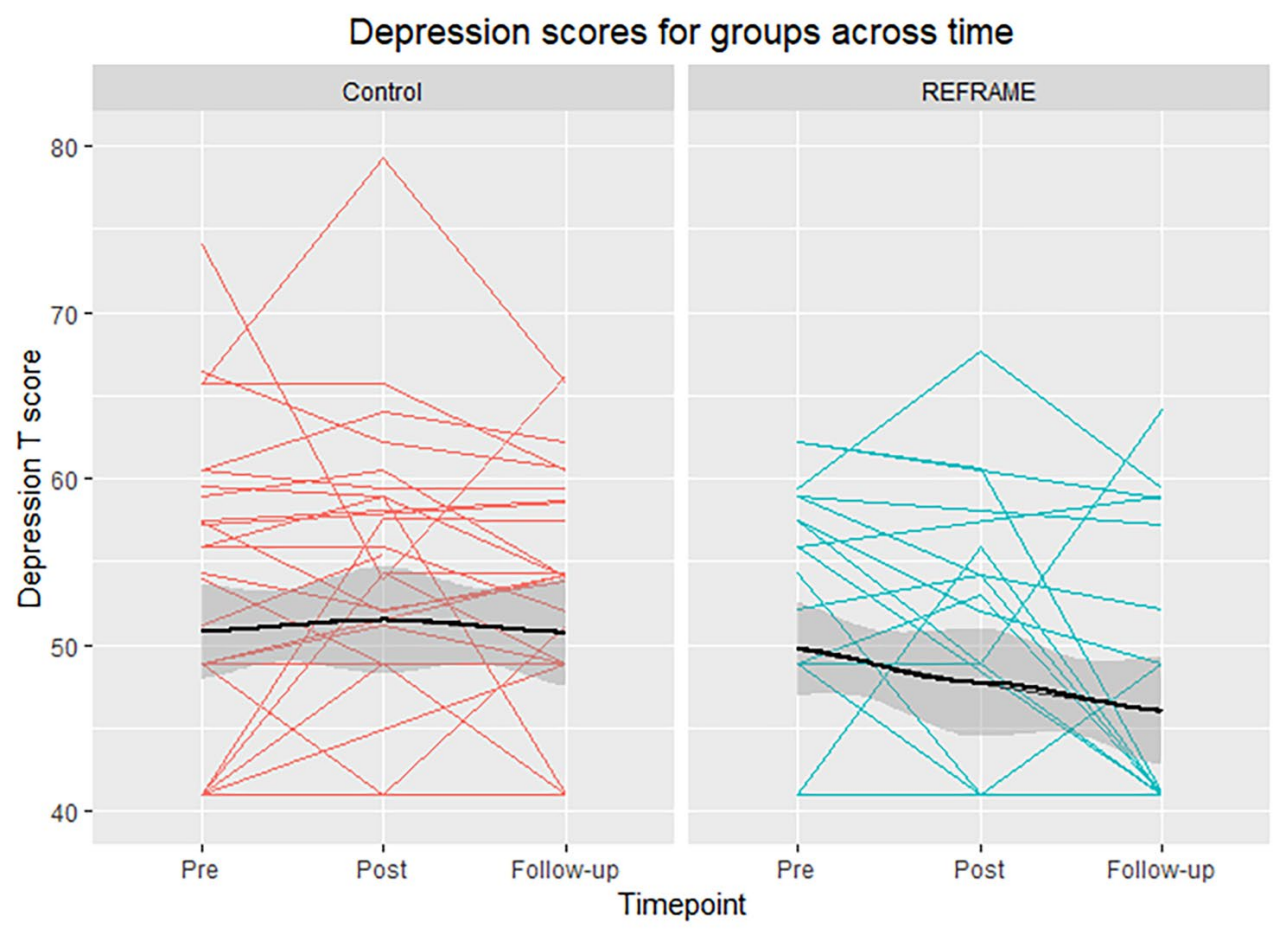
Graph of individual depression T scores for each participant across the three timepoints from pre- to post-intervention, and follow-up in Control (red) and REFRAME (blue) groups. Colored lines represent individual participant scores. Black lines represent group means, with surrounding gray representing the LOESS smooth curve with 95% confidence interval

For depression, a significant group-time interaction was found at follow-up (B = -3.52, *p* = .050, 95% CI = -6.97, − 0.08). Depression scores were lower in the REFRAME group (46.0 ± 7.9) than the control group at follow-up (50.7 ± 8.4; Fig. 4). Relative to baseline, depression scores were significantly reduced at follow-up in the time only (reduced) model (B = -1.87, *p* = .037). On average, scores decreased by 1.6 points from baseline to follow-up. These effects remained when Coronavirus anxiety was added (Table S2).

Well-being scores increased regardless of group in the model that included Coronavirus anxiety as a covariate (B = 0.60, *p* = .006, 95% CI = 0.17, 1.02; Table S2, Appendix 2). On average, well-being scores increased by 0.4 points from baseline to follow-up.

***Participant feedback.*** Overall, participants found the modules easy to follow and the session length to be manageable. Some participants expressed that the material was repetitive in parts, as well as a desire to receive feedback on the exercises. The three aspects of the intervention that participants found most helpful were learning to face their fears about memory loss and normalizing them, receiving educational information on memory loss and ADRD, and mindfulness and relaxation exercises.

## Discussion

To our knowledge, this is the first study to investigate the efficacy of a brief psychological intervention to promote adaptive coping in older adults experiencing heightened fear and avoidance specific to memory loss. Our intervention included three core components: psycho-education around ADRD, training in mindful awareness of fears, and behavioral activation [25]. We hypothesized that behavioral activation would be particularly effective in addressing maladaptive patterns of avoidant coping, such as social withdrawal and reduced cognitive exertion due to fear. As such, we employed an active control group who received psychoeducation and mindfulness only. This allowed us to compare potential benefits of addressing fearful thoughts and behavioral avoidance separately.

As hypothesized, both REFRAME and active control intervention arms were associated with a reduction in fears and avoidance behaviors around the ADRD symptom of memory loss. Specifically, we observed a significant reduction in fear-avoidance scores, which was maintained at 4-week follow-up. In addition to our primary outcome, we observed a significant decrease in fear of AD, which was also maintained at follow-up. These positive findings for both primary and secondary fear-based outcomes indicate that providing psycho-education about ADRD and tailored mindfulness exercises is beneficial for individuals experiencing heightened fear.

Contrary to our original hypothesis, effects on fear reduction were not stronger in the REFRAME compared to the control group. Both REFRAME and control groups included guided mindfulness exercises intended to impact the way participants interpreted everyday memory lapses. Specifically, participants were trained to be aware of distressing thoughts and feelings (e.g., *“memory lapses mean I’m unwell”*) without fixating on them. These exercises may have helped participants to notice everyday memory processes with greater objectivity rather than with a sense of fearful intensity [39]. As hypothesized, the frequency of self-reported memory failures decreased from baseline to follow-up in both groups. Two mechanisms of change may explain this outcome. First, shifting from a fearful to a more mindful self-perspective may have resulted in less vigilance around memory lapses, leading to a decrease in reported lapses. Second, psycho-education and mindfulness-based fear exercises may have reduced cognitive load, thereby reducing the frequency of actual lapses. Importantly, our intervention did not lead to an increase in memory lapses, which would be expected if participants had become more fixated on their memory.

Anxiety and depression symptoms decreased over time, while ability to participate in social activities and well-being increased. As hypothesized, the REFRAME condition was associated with a greater reduction in depression scores relative to the control condition, implying that the inclusion of therapeutic components to boost cognitive and social engagement (i.e., behavioral activation) had a unique positive impact. This finding is consistent with our etiological model, which suggests that poorer health and well-being outcomes in older adults’ results, at least partially, from avoidant coping strategies intended to mitigate distress around perceived changes in memory ability [16]. These results are also in keeping with the broader mental health literature. For example, behavioral activation is an evidence-based psychological treatment for depression [40]. On the other hand, behavioral avoidance is thought to contribute to depression onset and maintenance by limiting opportunities for positive experiences and reinforcing negative information biases that increase vulnerability [41].

Overall, our findings demonstrate that a low-intensity psychological intervention has salutary effects in older adults who are fearful and avoidant of ADRD, and that these benefits extend to broader health-related outcomes. That fears and avoidance behaviors can be mitigated is important because ADRD are among the most feared conditions associated with aging [2, 42]. As such, effective management of these fears has real-world implications for lifestyle risk reduction efforts. For example, promoting adaptive coping with ADRD-related fears may encourage individuals to maintain engagement in activities that are protective against ADRD but were previously avoided out of fear.

Identifying strategies to manage fear and avoidance coping associated with ADRD will become more of a priority with the advent of second-generation memory clinics [43], whose target population includes the so-called “worried well”, and as the conversation around brain health expands to even younger adult populations [44]. In addition, direct-to-consumer products are increasing access to genetic testing, making it easier for anyone to learn their APOE status [45]. The inevitable increase in access to genetic risk factors is already raising ethical considerations [46, 47]. An important outcome will therefore be to ensure that fears can be channeled into adaptive behaviors, such as help-seeking and care plan formulation.

Like other health domains, the relationship between fear, behavioral avoidance and lifestyle risk factors is likely to be reciprocal and recursive [48]. Thus, addressing fear and avoidance early on may accrue downstream benefits. For example, middle age is increasingly being viewed as a critical window for intervention, before the accumulation of significant brain pathology [49]. The ability to modify fear-avoidance patterns demonstrated here in older adults may also apply to younger, middle-aged groups. Addressing fear in this way could be a promising low-cost strategy, which could be incorporated as part of multi-domain interventions aimed at preventing ADRD.

Our study had multiple strengths. To our knowledge, this study is the first to evaluate the efficacy of a psychological intervention to promote adaptive coping in healthy older adults experiencing heightened fears and avoidance behaviors specific to the ADRD symptom of memory loss. The use of an RCT design with an active control condition allowed us to compare fear and avoidance components separately. Our sample size was sufficiently powered to detect medium-sized within- and between-groups differences. Our intervention proved feasible to deliver (> 75% module completion). Finally, inclusion of multiple measures and time points allowed for comprehensive outcome assessment.

One limitation was the short length of follow-up. Four weeks may have been too short to gauge efficacy around broader lifestyle-related outcomes. However, as this study involved a novel intervention, our primary aim was to demonstrate initial evidence of efficacy and proof-of-concept. Another limitation was that remote delivery required participants to have a good level of digital literacy, which may have biased the sample. The sample was predominately White, female, and well-educated, which limits generalizability. These limitations will be addressed in a larger-scale RCT with a more diverse sample, currently under development. Replication in a larger trial will also allow for more in-depth analysis of attrition and retention, and potential factors driving these. Finally, our intervention did not assess reasons underlying individuals’ fear of memory loss. Future work is needed to better understand these root causes in order to address the pervasive societal stigma surrounding ADRD.

Extensions of this work should include a larger RCT with a more diverse sample and longer follow up, or ‘booster’ sessions, to further evaluate the efficacy of the intervention. An important avenue for future research will be to focus on higher risk groups, such as people with family history of ADRD, APOE4 carriers, family carers, or clinical populations (e.g., individuals attending memory clinics). Contrary to our original hypothesis that outcome effects would be stronger in the REFRAME group, we observed only one significant difference for depression as a secondary outcome. This was likely due to the use of an active comparison. Future studies should therefore consider if increasing content or duration would lead to stronger group differences. This will be particularly important for teasing apart effects of psychological fear and behavioral avoidance components. Finally, future studies could consider a more individualized intervention approach, consistent with precision medicine frameworks [50]. This may involve tailoring exercises to individual fears or avoidance behaviors or the use of ecological momentary assessment tools to capture ‘in the moment’ fears.

## Conclusions

In conclusion, our study evaluated the efficacy of a psychological intervention to reduce ADRD-specific fear and avoidance behaviors in older adults who were highly fearful, but who did not exhibit cognitive impairment. Our intervention was effective at reducing fear and avoidance of memory loss, fear of AD, self-reported memory failures, anxiety, and depression symptoms, and at increasing ability to participate in social activities and well-being. Salutary outcomes were found for both groups, suggesting that psycho-education and mindfulness exercises were sufficient to demonstrate the interventions’ effectiveness. However, including therapeutic components to disrupt patterns of avoidant coping had additional positive outcomes on mood and affect, suggesting a key role for behavioral activation.

### Electronic supplementary material

Below is the link to the electronic supplementary material.

Supplementary Material 1

## Data Availability

The datasets used and analyzed during the current study are available from the corresponding author on reasonable request.

## Abbreviations

AD: Alzheimer’s disease
ADRD: Alzheimer’s disease and related dementias
ANOVA: Analysis of variance
APOE4: Apolipoprotein E4
CAS: Coronavirus Anxiety Scale
FADS: Fear of Alzheimer’s Disease Scale
FAM: Fear and Avoidance of Memory Loss scale
GDS-15: Geriatric Depression Scale
MFS: Memory Failure Scale
MoCA-Blind: Montreal Cognitive Assessment for blind individuals
PROMIS-29: Patient-Reported Outcomes Measurement Information System Profile
REFRAME: Reducing fear and avoidance of memory loss
RCT: Randomized control trial
WHO-5: World Health Organization-Five Well-Being Index
