# Supplementary material for: Reducing fear and avoidance of memory loss improves mood and social engagement in community-based older adults: a randomized trial
Source: BMC Geriatr. 2023 Nov 29;23:786. doi: 10.1186/s12877-023-04470-4 (PMC10688470; doi:10.1186/s12877-023-04470-4)
Supplement: Supplementary file 1 — Supplementary Material 1 [file 12877_2023_4470_MOESM1_ESM.docx]

**Appendices**

**Appendix 1**

**Intervention program**

Participants completed the intervention across three weeks. REFRAME and active control groups received the same intervention on Weeks 1 and 2, as follows. Week 1 consisted of psychoeducation, specifically:

- Introduction to ADRD; definitions and core symptoms
- Definitions of memory lapses and memory loss; differences between normal age-related forgetting and more serious memory problems
- Introduction to the concepts of general health anxiety and health anxiety specific to ADRD
- Prevalence of ADRD-specific fear in the general population

Week 2 consisted of fear-based mindfulness exercises, specifically:

- Introduction to the concept of mindfulness
- Introduction to basic mindfulness-based exercises, such as the body scan
- Guided meditations tailored to ADRD-specific fear.

REFRAME and active control groups received different intervention materials on Week 3. For the active control group, Week 3 consisted of continued mindfulness; specifically, introduction to new mindfulness exercises and guided meditations. For the REFRAME group, Week 3 consisted of behavioral activation exercises, specifically:

- Introduction to the concepts of avoidance, safety behaviors and behavioral activation
- Introduction to strategies for overcoming behavioral avoidance in the context of ADRD-specific fear.
- Guided exercises to increase awareness of avoidance patterns and identify strategies to challenge them.

Each week comprised 4-5 individual modules lasting approximately 30 minutes. Participants received a weblink to the intervention materials at the beginning of each week and were free to complete the modules in one sitting or spread out, depending on their preference. Each module included a combination of written and audio exercises. Full details of the intervention have been published previously.^15^ The trial was also registered on ClinicalTrials.gov (ID = NCT04821960, date: 30/03/2021) at: <https://clinicaltrials.gov/ct2/show/NCT04821960>.

**Appendix 2**

No significant differences were found between completers and non-completers in the REFRAME (*p*s > .168) or active control groups (*p*s > .081).

**Table S1**

*Participant Demographics by Group and Completion.*

|  | **REFRAME (n = 40)** | | **Control (n = 41)** | | **Total (n = 81)** | |
| --- | --- | --- | --- | --- | --- | --- |
|  | **Completers**  **(n = 30)** | **Non-completers**  **(n = 10)** | **Completers**  **(n = 36)** | **Non-completers**  **(n = 5)** | **Completers (n = 66)** | **Non-completers**  **(n = 15)** |
| Demographics |  |  |  |  |  |  |
| Age (years ± SD) | 64.3 (6.8) | 62.1 (6.9) | 66.9 (6.9) | 68.1 (9.4) | 65.8 (6.9) | 63.6 (8.0) |
| Gender (women, %) | 23 (76.7) | 6 (60.0) | 27 (75.0) | 2 (40.0) | 49 (75.4) | 9 (56.3) |
| Race (White, %) | 24 (80.0) | 8 (80.0) | 35 (97.2) | 4 (80.0) | 59 (90.8) | 12 (75.0) |
| Ethnicity (non-Hispanic, %) | 29 (96.7) | 10 (100) | 36 (100) | 5 (100) | 64 (98.5) | 16 (100) |
| Education (college degree or higher, %) | 19 (63.3) | 7 (70.0) | 32 (88.9) | 3 (60.0) | 51 (78.5) | 10 (62.5) |
| Employment (retired, %) | 15 (50.0) | 4 (40.0) | 19 (52.8) | 2 (40.0) | 34 (52.3) | 6 (37.5) |
| Income (high, %) | 18 (60.0) | 5 (50.0) | 19 (52.8) | 4 (80.0) | 25 (39.1) | 5 (33.3) |
| Family history of ADRD (yes, %) | 14 (46.7) | 5 (50.0) | 19 (52.8) | 0 (0) | 32 (50.7) | 5 (31.3) |
| MoCA-Blind (mean ± SD) | 19.7 (1.1) | 19.7 (1.5) | 19.9 (1.3) | 19.2 (1.3) | 19.9 (1.2) | 19.4 (1.4) |
| Depression (GDS-15; mean ± SD) | 3.3 (2.4) | 3.3 (2.7) | 3.8 (2.8) | 3.2 (2.4) | 3.6 (2.6) | 3.1 (2.5) |
| Fear and avoidance of memory loss (mean ± SD) | 68.4 (5.1) | 66.2 (6.4) | 68.4 (5.1) | 65.8 (5.8) | 68.4 (5.1) | 66.1 (5.8) |
| Fear of Alzheimer’s disease (mean ± SD) | 43.9 (21.2) | 52.8 (8.1) | 48.7 (21.2) | 37.0 (15.4) | 46.6 (21.2) | 44.9 (14.3) |
| Memory failures (mean ± SD) | 32.1 (9.0) | 32.8 (5.9) | 34.8 (8.1) | 35.8 (5.3) | 33.6 (8.5) | 34.3 (5.5) |
| Patient-reported outcomes scales |  |  |  |  |  |  |
| Anxiety (mean ± SD) | 8.4 (3.5) | 8.4 (2.7) | 8.4 (2.7) | 6.2 (2.6) | 54.8 (7.6) | 52.5 (7.5) |
| Depression (mean ± SD) | 6.3 (2.7) | 7.6 (3.8) | 7.3 (3.7) | 4.8 (1.8) | 49.8 (8.7) | 47.9 (8.8) |
| Social function (mean ± SD) | 15.5 (4.1) | 15.4 (3.1) | 15.1 (3.8) | 18.0 (1.8) | 53.3 (8.3) | 54.6 (6.7) |
| Well-being (mean ± SD) | 14.0 (6.2) | 15.0 (3.9) | 14.0 (5.8) | 13.6 (5.6) | 14 (5.9) | 14.3 (4.6) |

Notes: Social function refers to participants’ ability to participate in social activities and roles. Abbreviations: ADRD = Alzheimer’s disease and related dementias; MoCA-Blind = Montreal Cognitive Assessment for visually impaired individuals.

**Table S2**

*Regression models with Coronavirus anxiety scores included as a covariate.*

|  | **FAM** | | **FADS** | | **MFS** | | **Anxiety** | | **Depression** | | **Social function** | | | **Well-being** | |
| --- | --- | --- | --- | --- | --- | --- | --- | --- | --- | --- | --- | --- | --- | --- | --- |
|  | **B** | ***p*** | **B** | ***p*** | **B** | ***p*** | **B** | ***p*** | **B** | ***p*** | **B** | ***p*** | **B** | | ***p*** |
| Intercept | 78.63 | *<*.001*** | 80.98 | *<*.001*** | 26.81 | .002** | 71.92 | *<*.001*** | 56.69 | *<*.001*** | 52.75 | *<*.001*** | 1.49 | | .79 |
| Age | -.17 | .231 | -.52 | .099 | .10 | .406 | -.26 | .020* | -.09 | .487 | -.01 | .976 | .19 | | .030* |
| CAS | .13 | .391 | .13 | .692 | .03 | .789 | .17 | .242 | .17 | .306 | -.19 | .250 | -.07 | | .447 |
| Week 1 | -1.9 | .001*** | - | - | - | - | - | - | - | - | - | - | - | | - |
| Week 2 | -4.2 | *<*.001*** | - | - | - | - | - | - | - | - | - | - | - | | - |
| Week 3 | -5.6 | *<*.001*** | - | - | - | - | - | - | - | - | - | - | - | | - |
| Post | -6.5 | *<*.001*** | -4.35 | *<*.001*** | -.83 | .008** | .05 | .904 | .89 | .142 | 1.72 | *<*.001*** | - | | - |
| Follow-up | -6.3 | *<*.001*** | -5.43 | *<*.001*** | -1.69 | *<*.001*** | -1.9 | *<*.001*** | -.60 | .321 | 2.22 | *<*.001*** | .60 | | .006** |
| Week 3*group | - | - | - | - | - | - | - | - | -3.3 | *<*.001*** | - | - | - | | - |

Notes: CAS = Coronavirus Anxiety Scale; FAM = Fear and Avoidance of Memory Loss scale; FADS = Fear of Alzheimer’s disease Scale; MFS = Memory Failures Scale. Social function refers to participants’ ability to participate in social roles and activities. ****p* < .001; ***p* < .01; **p* < .05. Estimates are relative to baseline.
